# A Novel In-Cell ELISA Assay Allows Rapid and Automated Quantification of SARS-CoV-2 to Analyze Neutralizing Antibodies and Antiviral Compounds

**DOI:** 10.3389/fimmu.2020.573526

**Published:** 2020-10-09

**Authors:** Lara Schöler, Vu Thuy Khanh Le-Trilling, Mareike Eilbrecht, Denise Mennerich, Olympia E. Anastasiou, Adalbert Krawczyk, Anke Herrmann, Ulf Dittmer, Mirko Trilling

**Affiliations:** ^1^ Institute for Virology, University Hospital Essen, University of Duisburg-Essen, Essen, Germany; ^2^ Department of Infectious Diseases, West German Centre of Infectious Diseases, University Hospital Essen, University of Duisburg-Essen, Essen, Germany

**Keywords:** SARS-CoV-2, COVID-19, neutralizing, antibodies, vaccine, interferon

## Abstract

The coronavirus disease 2019 (COVID-19) caused by the severe acute respiratory syndrome coronavirus 2 (SARS-CoV-2) is currently the most pressing medical and socioeconomic challenge. Constituting important correlates of protection, the determination of virus-neutralizing antibodies (NAbs) is indispensable for convalescent plasma selection, vaccine candidate evaluation, and immunity certificates. In contrast to standard serological ELISAs, plaque reduction neutralization tests (PRNTs) are laborious, time-consuming, expensive, and restricted to specialized laboratories. To replace microscopic counting-based SARS-CoV-2 PRNTs by a novel assay exempt from genetically modified viruses, which are inapplicable in most diagnostics departments, we established a simple, rapid, and automated SARS-CoV-2 neutralization assay employing an in-cell ELISA (icELISA) approach. After optimization of various parameters such as virus-specific antibodies, cell lines, virus doses, and duration of infection, SARS-CoV-2-infected cells became amenable as direct antigen source for quantitative icELISA. Antiviral agents such as human sera containing NAbs or antiviral interferons dose dependently reduced the SARS-CoV-2-specific signal. Applying increased infectious doses, the icELISA-based neutralization test (icNT) was superior to PRNT in discriminating convalescent sera with high from those with intermediate neutralizing capacities. In addition, the icNT was found to be specific, discriminating between SARS-CoV-2-specific NAbs and those raised against other coronaviruses. Altogether, the SARS-CoV-2 icELISA test allows rapid (<48 h in total, read-out in seconds) and automated quantification of virus infection in cell culture to evaluate the efficacy of NAbs and antiviral drugs using reagents and equipment present in most routine diagnostics departments.

## Highlights

Knowledge concerning SARS-CoV-2-neutralizing antibodies (NAbs) is indispensable for COVID-19 convalescent plasma selection, evaluation of vaccines, and immunity certificates. Our in-cell ELISA (icELISA) test allows rapid (<48 h) and high-throughput detection and quantification of SARS-CoV-2-specific NAbs and antiviral activity of drug candidates.

## Introduction

By the time of writing, more than 29 million people experienced a laboratory confirmed infection with the severe acute respiratory syndrome coronavirus (SARS-CoV)-2 and more than 930,000 people died while having coronavirus infectious disease 19 (COVID-19). First surveillance studies and calculations of excess mortality rates indicate that the precise number of infections and the true number of fatalities exceed above-mentioned numbers by far.

Coronaviruses (CoVs) are positive strand RNA viruses widespread among various vertebrate hosts including bats and rodents (1). Together with four seasonal human CoVs (HCoVs) and the two other emerging HCoVs SARS-CoV-1 and MERS-CoV, SARS-CoV-2 is the seventh HCoV causing widespread human diseases (2). In December 2019, SARS-CoV-2 was first recognized in the Hubei province in China (3) from where it rapidly spread throughout the world. In addition to its genetic similarity, SARS-CoV-2 not only shares some clinical characteristics with SARS-CoV-1 (4) but also exhibits some highly relevant particularities such as an increased spreading efficacy and the length of the course of disease (5). On January 31, the WHO declared the SARS-CoV-2 outbreak a Public Health Emergency of International Concern. On March 11, 2020, WHO started to denote the outbreak a global pandemic. The center of the pandemic shifts between regions, posing danger of repetitive local and temporal reintroduction circles. Thus, even countries that managed the first wave must prepare in terms of diagnostics capacities for potential future re-emergences.

Most SARS-CoV-2 infections lead to mild or moderate illnesses. However, a considerable fraction of cases proceeds to severe pneumonia or life-threatening acute respiratory distress syndrome. Elderly individuals and people with pre-existing comorbidities such as impaired immunity, chronic respiratory diseases, cardiovascular diseases, and cancer are more prone to suffer from severe COVID-19. The case fatality rate (CFR) is difficult to calculate in the midst of the pandemic. Depending on the age, CFR estimates of up to 18.4% for individuals older than 80 years and 1.38% (range 1.23–1.53) for the general population have been reported (6).

Given the extent, pace, and severity of the COVID-19 pandemic, diagnostics departments even in countries with highly developed medical systems struggle to provide sufficient and timely test capacities. Since nucleic acid-based pathogen detection has a very short window of opportunity, assays detecting long-lasting immune responses such as antibodies are required to monitor virus spread and to estimate potential herd immunity.

With some delay, most infected individuals raise a detectable humoral immune response including specific immunoglobulins (Ig) M, IgA, and IgG (7–9). Neutralizing antibodies (NAbs) bind and abrogate the function of viral proteins such as the SARS-CoV-2 Spike (S) protein that are essential for virus entry into host cells, e.g., through recognition of the cognate receptor ACE2 (10). Accordingly, monoclonal NAbs exhibit strong therapeutic and prophylactic efficacies in SARS-CoV-2-infected human ACE2-transgenic mice (11). A recent vaccination study conducted in non-human primates identified NAbs as correlate of protection (12), indicating that a human SARS-CoV-2 vaccine should also be capable to elicit potent NAb responses. Accordingly, first human vaccine studies focussed on the question if NAbs were induced (13). Additionally, monoclonal NAbs and NAb-containing hyper-immunoglobulin preparations may be applicable as treatment against COVID-19 (14). NAbs are also the backbone of convalescent plasma (CP) therapies (15–17) that are one of seven clinical recommendations of the IDSA (18). Based on the havoc COVID-19 causes to the global economy, immunity passports and vaccination certificates, documenting protection through NAbs, have been discussed [e.g., (19)]. Taken together, SARS-CoV-2-specific NAbs and their quantification appear to be of central importance for the medical and socio-economic management of the pandemic.

Different types of neutralization tests (NT) have been developed for SARS-CoV-2. However, to our knowledge, these assays rely on laborious microscopic counting of virus plaques or antibody-stained foci by trained personnel (20–22) or on genetically modified viruses such as transgenic SARS-CoV-2 mutants (23) or pseudo-typed viruses [e.g., vesicular stomatitis virus (VSV) or human immunodeficiency virus (HIV)] expressing the S protein of SARS-CoV-2 (24, 25). Genetically modified viruses are generally prohibited in routine diagnostics laboratories and usually not applicable in less developed regions. Due to the central importance of NAbs and the limitations of the currently available methodology, we established a cheap, simple, fast, reliable, and automatable in-cell ELISA (icELISA)-based icNT applicable in routine diagnostics departments with access to BSL3 laboratories.

## Materials and Methods

### Cells, Viruses, Interferons, and Sera

Caco-2 (ATCC HTB-37) and Vero E6 (ATCC CRL-1586) were cultivated in Roswell Park Memorial Institute 1640 [RPMI-1640 (Gibco 21875-034)] and high glucose Dulbecco`s minimal essential medium [DMEM (Gibco 41966-029)], respectively, supplemented with 10% (v/v) FCS, penicillin, and streptomycin at 37°C in an atmosphere of 5% CO_2_. SARS-CoV-2 was isolated from a patient sample using Vero E6 and confirmed by SARS-CoV-2 diagnostic qRT-PCR. Viral titers were determined by TCID50 titration. Human IFNα2 and IFNβ were purchased from PBL Assay Science (#11101) and Peprotech (#300-02BC), respectively. The collection of serum samples and the virus isolation have been approved by the ethics committee of the medical faculty of the University of Duisburg-Essen (20-9208-BO, 20-9511-BO, and 20-9512-BO). Anti-SARS-CoV-2 IgG antibodies were detected using the ELISA detecting antibodies recognizing the SARS-CoV-2 spike protein (Euroimmun Medizinische Labordiagnostika, Germany) according to manufacturer’s instructions.

### SARS-CoV-2 icELISA and icNT

**Figure 1:**
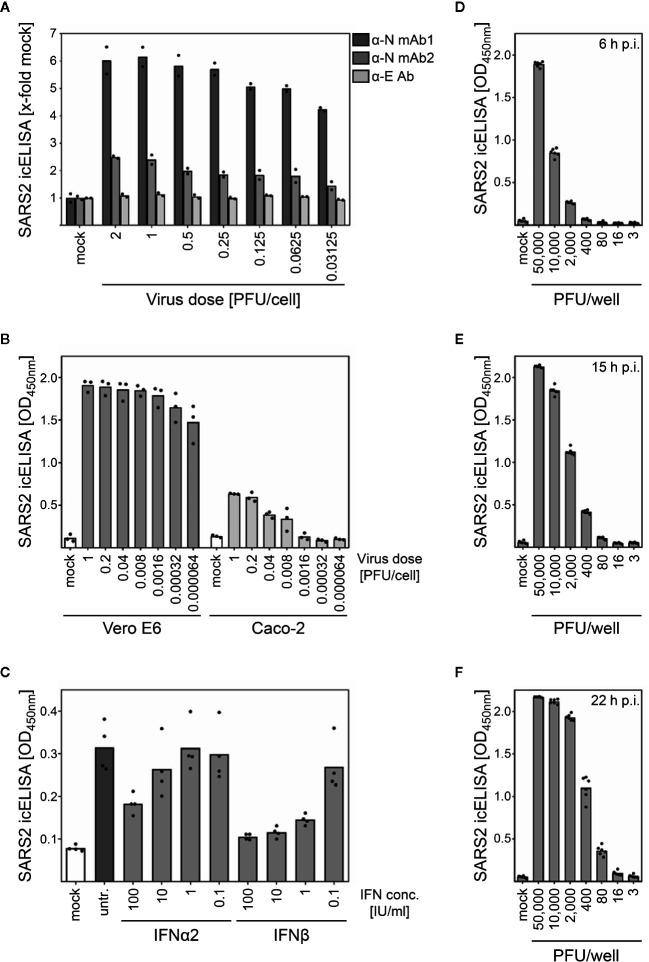
The icELISA test allows quantification of SARS-CoV-2 replication and its inhibition by antiviral compounds. **(A)** Caco-2 cells were infected with indicated doses of SARS-CoV-2. At 3 d p.i., cells were fixed and detected by icELISA using E- and N-specific primary antibodies. For all further icELISAs, α-N mAb1 was used. **(B)** Vero E6 and Caco-2 cells were infected with indicated doses of SARS-CoV-2. At 3 d p.i., cells were analyzed by icELISA. **(C)** Caco-2 cells were treated with indicated concentrations of IFNα2 or IFNβ. At 3 h post treatment, cells were infected with SARS-CoV-2 (MOI 0.1). Viral replication was evaluated at 3 d p.i. by icELISA. **(D–F)** Vero E6 cells were infected with indicated doses of SARS-CoV-2. At 6, 15, and 22 h p.i. (D, E, and F, respectively), and cells were analyzed by icELISA. Bars depict the mean values. Dots show the values of the individual measurements.

For the analysis of neutralizing antibodies, serum samples were inactivated at 56°C for 30 min. A detailed icELISA/icNT protocol is provided in **Supplementary File 1**. Briefly, defined doses of SARS-CoV-2 were incubated with different serum dilutions for 1 h at 37°C prior to Vero E6 infection. At 16-24 h p. i., cells (~5 × 10^4^/well of a 96-well plate) were fixed with 4% (w/v) paraformaldehyde/PBS. Cells were permeabilized with 1% (v/v) Triton-X-100/PBS and blocked with 3% (v/v) FCS/PBS. The primary antibody was added and incubated for 2 h at room temperature or overnight at 4°C. Peroxidase-labelled secondary antibody was incubated for 1–2 h. Washing steps were performed with 0.05% (v/v) Tween-20/PBS. Tetramethylbenzidin (TMB) substrate was added to visualize the enzyme reaction. The reaction was stopped with 0.5 M HCl. Subsequently, the absorbance was measured using a microplate multireader (Mithras2 LB 943; Berthold Technologies). The α-N mAb1 (ABIN6952435), α-N mAb2 (ABIN6952433), α-E Ab (ABIN1031551), and POD-coupled secondary antibodies (Dianova) were used. In the case that an absolute quantification is necessary, the relative quantification of the icELISA can be transformed into an absolute quantification in terms of the virus dose using virus calibration curves (like in **Figure 1**) or using commercially available recombinant N preparations.

### Immunoblot Analysis

Immunoblotting was performed as described previously (26) using antibodies recognizing the SARS-CoV-2 N protein (ABIN6952435) or GAPDH (sc-25778, Santa Cruz). Lysates were inactivated by sequential heat incubation (10 min at 70°C and 10 min at 95°C) before they were discharged from the BSL3 laboratory. Proteins were visualized using peroxidase-coupled secondary antibodies (Dianova) and the ECL chemiluminescence system (Cell Signaling Technology).

## Results

### Relative Quantification of SARS-CoV-2 Replication and its Inhibition by Antiviral Compounds Using icELISA

We hypothesized that virus-encoded proteins expressed by infected cells should be amenable as source of viral antigens for the detection and quantification by ELISA. We optimized the experimental conditions such as cell type, virus dose, infection period, cell fixation method, blocking reagent, amd type and concentrations of primary and secondary antibodies (**Figure 1** and data not shown). As described in the Materials and Methods section and provided as detailed laboratory protocol in the supplementary information (**Supplementary File 1**), we compared different commercially available antibodies for the icELISA-based quantification of SARS-CoV-2 proteins. Based on existing data on virus entry (10), we infected human Caco-2 cells with graded virus doses (ranging from 0.03125 to 2 PFU/cell). At 3 days post infection (d p.i.), we fixed and stained the cells with different antibodies either recognizing the E or the N protein of SARS-CoV-2. In accordance with high expression level of the N protein (27, 28), certain N-specific mouse IgGs exhibited a signal-to-noise ratio favourable for icELISA (**Figure 1A**). A comparison of Vero E6 and human Caco-2 cells revealed that the icELISA is applicable to different cells (**Figure 1B**).

Since the icELISA signal directly correlated with viral replication and viral antigen expression, we tested its ability to determine antiviral effects. Human Caco-2 cells were treated with graded concentrations of human interferon (IFN) α2 or IFNβ and infected 3 h later with SARS-CoV-2. At 3 d p.i., viral antigen amounts were quantified by icELISA. In accordance with a recent clinical phase 2 trial (29), IFNβ exhibited strong and dose-dependent antiviral activity against SARS-CoV-2 in human cells (**Figure 1C**), indicating that the icELISA is applicable for future experiments addressing the efficacy of potential antiviral drugs in human cells.

**Figure 2:**
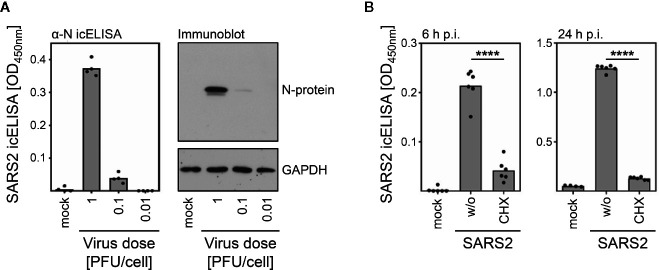
The icELISA faithfully reports on SARS-CoV-2 N protein expression. **(A)** Vero E6 cells were infected with indicated doses of SARS-CoV-2. At 6 h p.i., cells were fixed for icELISA analysis or lysed for immunoblot analysis. Bars depict icELISA mean values. Dots show values of individual measurements. Immunoblot analysis was performed using antibodies recognizing the N protein of SARS-CoV-2 or GAPDH. **(B)** Vero E6 cells were infected with SARS-CoV-2 (10,000 PFU per well) in the absence (‘w/o’) or presence of the translation inhibitor cycloheximide (‘CHX’). At 6 and 24 h p.i., cells were analyzed by icELISA. Bars depict mean values. Dots show values of individual measurements. Significance was calculated by unpaired two-tailed student´s t-test. ****p-value < 0.0001.

Despite different start MOIs, similar icELISA signals were observed at 72 h p.i. in Vero E6 (**Figure 1B**), indicating multiple rounds of virus amplification and extraordinary fast replication kinetics of SARS-CoV-2 in Vero E6 cells, consistent with previous studies (30). To test if shorter infection periods might result in virus dose-dependent signals, we analyzed infected Vero E6 cells after 6, 15, and 22 h. SARS-CoV-2 was readily detectable in Vero E6 cells by icELISA already at 6 h p.i. (**Figures 1D–F**). To evaluate if the icELISA faithfully reports on the amounts of SARS-CoV-2-expressed antigens, we infected cells with graded infectious doses and performed immunoblots and icELISAs in parallel. As expected, icELISA and immunoblot signals correlated very well (**Figure 2A**). Since we observed icELISA signals already at 6 h p.i. (**Figures 1A, D**) and the N protein is an abundant component of SARS-CoV-2 particles (31–33), the N protein derived from input viruses could have contributed to icELISA signals. To evaluate this, we infected cells in the presence and absence of the translation inhibitor cycloheximide (‘CHX’). Consistent with the notion that the icELISA signal is dominated by *de novo* N protein expression, the signal was significantly diminished by CHX (**Figure 2B**).

Taken together, these data indicate that the icELISA allowed rapid identification and relative quantification of SARS-CoV-2 replication in Vero E6 and human cells and its inhibition by antiviral compounds.

### Neutralization Tests Based On icELISA Allow the Quantification of SARS-CoV-2 NAbs as Early as 6 Hours Post Infection

**Figure 3:**
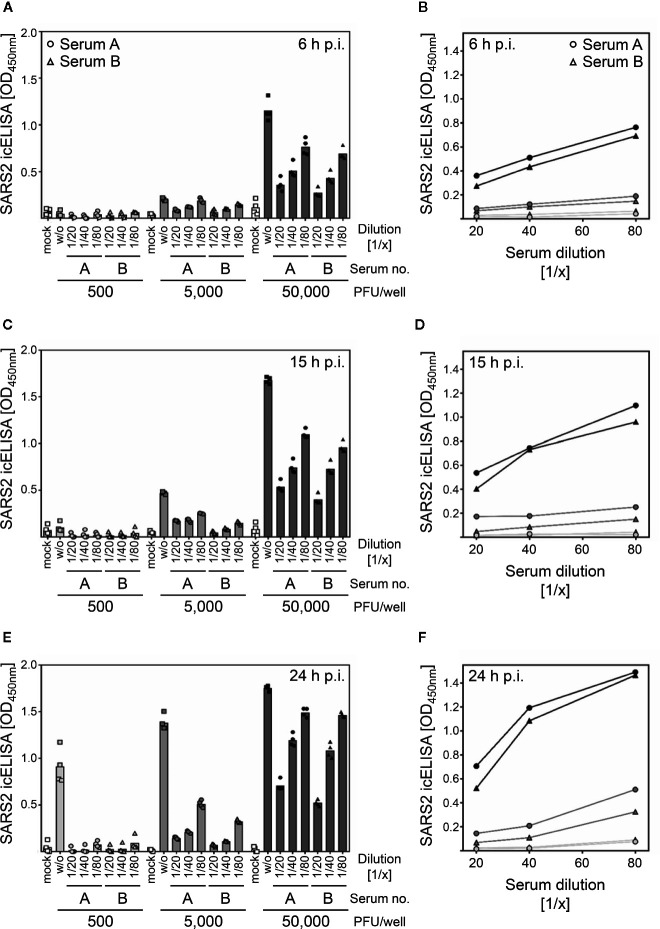
The icNT allows the quantification of SARS-CoV-2 NAbs as early as 6 h p.i. **(A–F)** 500, 5,000, and 50,000 PFU of SARS-CoV-2 were incubated with indicated dilutions of 2 convalescent sera for 1 h before Vero E6 cells were infected. At 6 h p.i. **(A, B)**, 15 h p.i. **(C, D)**, and 24 h p.i. **(E, F)**, cells were analyzed by icELISA to evaluate the neutralizing capacity of the sera. **(A, C, E)** Bars depict the mean values. Squares, dots, and triangles show the values of the individual measurements. **(B, D, F)** All mean values of the different serum dilutions and virus doses were depicted in one diagram to compare the influence of the input virus amount on the course of the curve. Light gray, 500 PFU. Gray, 5,000 PFU. Dark gray, 50,000 PFU.

Since the icELISA allowed simple and automated quantification of SARS-CoV-2-dependent antigen expression, we tested if an icELISA-based neutralization test (icNT) can be established. We infected Vero E6 cells for 6, 15, and 24 h with graded SARS-CoV-2 infection doses (500, 5,000, or 50,000 PFU/well) in the absence or presence of two convalescent sera in three different concentrations (1/20, 1/40, and 1/80 dilution). Using the high infectious dose, viral antigens became dose dependently detectable by icELISA as early as 6 h p.i. (**Figure 3A**). Both immune sera strongly and dose-dependently neutralized the virus-induced signal (**Figure 3B**). At 15 h p.i., the intermediate infectious virus dose also became detectable (**Figure 3C**) and both human sera efficiently neutralized the infection (**Figure 3D**). At 24 h p.i., all infection conditions resulted in a strong icELISA signal (**Figure 3E**), indicating that infectious doses as low as 500 PFU/well are detectable by icELISA after 24 h of infection. Even the strong icELISA signal at 24 h p.i. was dose dependently neutralized by both sera (**Figure 3F**). Please note that the neutralizing capacity of given sera dilutions was less pronounced at higher virus doses as compared to lower virus doses, as indicated by residual icELISA signals.

Taken together, the icELISA resulted in a time- and virus dose-dependent signal constituting a surrogate for SARS-CoV-2 infection and replication. The fact that the infection and the resulting icELISA signal were neutralized by NAbs present in immune sera indicated that the fast and automated icELISA format is applicable for icNTs.

### The icNT Results Correlate With the Standard SARS-Cov-2 Neutralization Test Conducted by Staining of Virus Foci and Microscopic Counting

**Figure 4:**
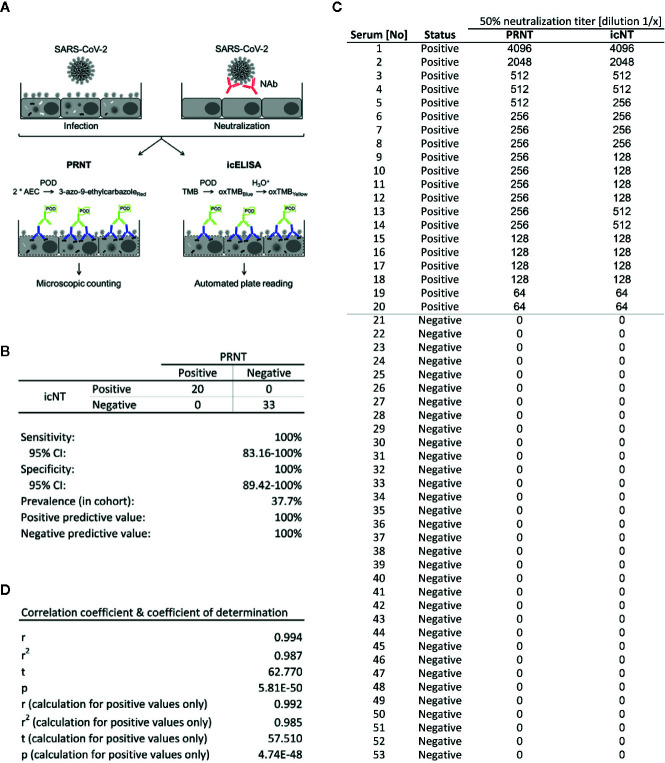
The icNT results correlate with the standard SARS-CoV-2 neutralization test. **(A)** Schematic representation of AEC stain-based classic NT and icELISA-based icNT. In the upper right panel, the intracellular viral antigens are missing due to the neutralization. NAb, neutralizing antibody. PRNT, plaque reduction neutralization test. POD, peroxidase. AEC, 3-amino-9-ethylcarbazole. TMB, tetramethylbenzidine. **(B)** Sensitivity and specificity calculation of icNT compared to the conventional PRNT. Calculations were performed using algorithms online available at https://www.medcalc.org/calc/diagnostic_test.php. **(C)** 200 PFU of SARS-CoV-2 were incubated with different dilutions of serum samples for 1 h before Vero E6 were infected. At 20 and 48 h p.i., neutralizing capacity was evaluated by icELISA and AEC staining with subsequent microscopic counting, respectively. The highest dilution capable to neutralize 50% of input was determined and results of PRNT and icNT were compared. The presence of detectable SARS2 NAbs (column: Status) and the 50% neutralization titers of PRNT and icNT are depicted. **(D)** Calculation of the correlation coefficient (r), the coefficient of determination (r^2^), and the p value for the entire data set shown in **(C)** (upper part) and the neutralizing samples (‘positive’) only (lower part).

Although most SARS-CoV-2 NTs have not been formally validated and certified, classic plaque reduction neutralization tests (PRNT) are currently considered to represent the gold standard for the detection of SARS-CoV-2-specific NAbs. Various commercially available IgM, IgA, and IgG ELISAs have been compared to PRNTs [e.g., (30)]. To validate the novel icNT, 53 sera—24 positive for SARS-CoV-2-specific IgG as determined using the Euroimmun ELISA (ELISA ratio: 1.13–9.92) and 29 ELISA-negative sera (ELISA ratio < 0.9)—were compared side-by-side by icNT and standard PRNT using 200 PFU/well (**Figure 4A**). One set was processed by icNT, while for the other set virus foci were stained using an antibody-based AEC staining method and manually counted by microscopy. Four ELISA-positive serum samples (ELISA ratios 1.13, 1.25, 3.31, and 4.06) consistently did not show neutralizing capacities in PRNT and icNT. The icNT correctly recognized all 20 neutralizing and all 33 non-neutralizing serum samples (**Figure 4B**), indicating optimal sensitivity [100%; 95% confidence interval (CI), 83.16–100%] and specificity (100%; 95% CI, 89.42–100%) in this restricted cohort (53 serum samples) purposefully selected to contain several positive specimens (37.7%). Additionally, we assessed the correlation between PRNT and icNT results (**Figure 4C**). Both methods gave almost congruent results in terms of the highest dilution resulting in 50% neutralization [Pearson’s correlation coefficient (r) = 0.994; coefficient of determination (r^2^) = 0.987; p value 5.81E-50]. Similar values were obtained, when we restricted the analysis to positive samples [Pearson’s correlation coefficient (r) = 0.992; coefficient of determination (r^2^) = 0.985; p value 4.75E-48] (**Figure 4D**), highlighting excellent test performances of the icNT compared to the PRNT.

### The icNT Discriminates SARS-CoV-2-Neutralizing Sera From Non-Immune Sera and Provides Superior Resolution When Increased Virus Doses are Used

**Figure 5:**
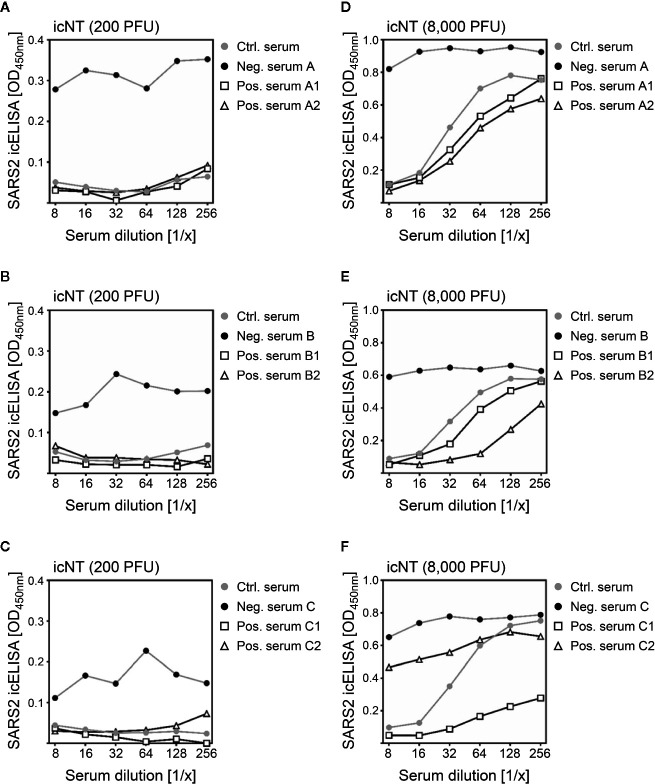
The icNT provides superior resolution upon usage of increased virus doses. **(A–F)** 200 or 8,000 PFU of SARS-CoV-2 were incubated with indicated dilutions of serum samples for 1 h before Vero E6 cells were infected. At 20 and 16 h p.i. (for 200 and 8,000 PFU, respectively), neutralizing capacity was evaluated by icELISA. Samples measured on the same plate are depicted in one diagram. The control serum was used as reference for all plates. Please note the different y-axis scales of the left and the right panel.

Standard SARS-CoV-2 NTs base on microscopic counting of plaques or antibody-stained virus foci. To enable plaque/foci recognition and individual counting by trained personnel, a countable number of PFU must be applied in PRNTs. Depending on the PRNT protocol, 100 (20) to 400 PFU (21) are used to infect each well. Based on previous experiences with virus neutralization experiments (34, 35), we suspected that lower infectious doses might be more susceptible to NAbs than higher virus doses, e.g., through altered ratios of NAbs and antigenic regions determining neutralization, such as the receptor binding domain (RBD) of the S protein of SARS-CoV-2. Accordingly, graded infectious doses (1,250–10,000 PFU per well) showed virus dose-dependent susceptibilities to the same serum sample (**Supplementary Figure S1**). Before we assessed clinical specimens, we compared icNT signals upon a high MOI infection with viral progeny as determined by parallel TCID50 titration. As expected, we observed that icNT signals and residual virus numbers correlated very well (**Supplementary Figure S2**). To address if the icNT can provide superior resolution in terms of discriminating intermediate from strong NAb responses in convalescent sera, we selected prototypical sera and conducted comparative icNTs applying either 200 PFU/well or 40-fold more virus (8,000 PFU/well). While all positive sera clearly neutralized the low infectious dose of 200 PFU/well (**Figures 5A–C**), the neutralizing capacity of the same sera considerably differed at the more restrictive high virus dose (**Figures 5D–F**). This advantage of the icNT compared to the PRNT was especially evident when a restricted number of dilutions was assessed. Despite their neutralizing capacity at low dose infections, some of the convalescent sera (e.g., serum C2) hardly show appreciable neutralization in high MOI infection and did not reach the typical benchmark of 50% neutralization at a 1/8 serum dilution. Thus, we concluded that the icNT principle allows higher infectious doses that appear to discriminate intermediate from superior SARS-CoV-2 neutralizing antibody titres.

### The SARS-CoV-2 icNT is Specific and Discriminates Between Serum Samples Either Recognizing SARS-CoV-2 or Other Coronaviruses

**Figure 6:**
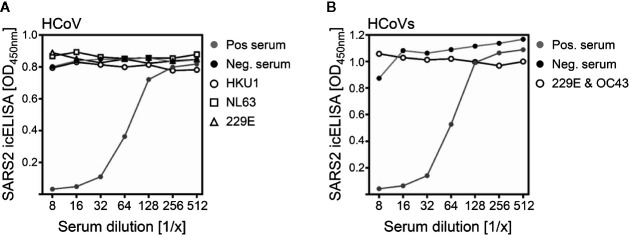
The SARS-CoV-2 icNT is specific and discriminates between serum samples either recognizing SARS-CoV-2 or other coronaviruses. **(A)** SARS-CoV-2 was incubated with indicated dilutions of serum samples for 1 h before Vero E6 cells were infected. Neutralizing capacity was evaluated by icELISA. Serum samples were obtained from individuals positively tested by diagnostic PCR (performed at the diagnostics department of the Institute for Virology of the University Hospital Essen) for HCoV-HKU1, HCoV-NL63, or HCoV-229E. Serum samples were taken at 2 to 3 months post symptom onset and positive PCR testing. **(B)** As in **(A)**, but a serum sample of an individual who experienced co-infection of two HCoVs was used. The individual was positively tested for HCoV-229E and HCoV-OC43.

In contrast to ELISAs or PCRs recognizing viral components in clinical specimens, NT test principles rely on intentional infections of cell cultures with well-defined virus preparations in the presence or absence of compounds that may impair the infection. Thus, the NT specificity results from the choice of virus applied to the cells. However, antiviral compounds can either be specific for one particular virus species (e.g., most monoclonal neutralizing antibodies), broadly active against virus families (e.g., receptor blocking agents) or even bigger genera (e.g., hyperimmunoglobulin preparations). In addition to SARS-CoV-2, other HCoVs such as the alphacoronaviruses HCoV-229E and HCoV-NL63 and the betacoronaviruses HCoV-OC43 and HCoV-HKU1 circulate autochthonously in the human population, resulting in high sero-prevalence rates (36). To address if the experimental SARS-CoV-2 infection in the icNT might be diminished by cross-reactive antibodies, we tested sera of individuals who have had infections with the alphacoronaviruses HCoV-229E or HCoV-NL63 or betacoronaviruses such as HCoV-HKU1 (**Figure 6A**) or a combination of HCoV-229E and HCoV-OC43 (**Figure 6B**). In contrast to the positive control containing SARS-CoV-2-specific NAbs, all tested serum samples recognizing other coronaviruses did not neutralize SARS-CoV-2. Although the limited sample size prevents definitive conclusions regarding cross-neutralizing capacities of antibodies raised by other HCoVs, in conjunction with consistent publications (37, 38) it indicates the specificity of the icNT assay.

## Discussion

We established a novel icELISA-based test principle for detection and relative quantification of SARS-CoV-2. Given the excellent signal-to-noise ratio between infected and uninfected cells, the test was applicable to quantify the efficacy of antiviral compounds, here shown for IFNβ, and SARS-CoV-2-specific NAbs present in immune sera.

Compared to icELISA and icNT, standard virus titrations and PRNTs are far more laborious, time consuming, and expensive—not to speak from subjective and expectation biases upon usage for research. The entire icNT can be processed in less than 2 days including the infection period. Given that the protocol includes an early fixation step using 4% (w/v) paraformaldehyde which inactivates SARS-CoV-2, all subsequent steps can be processed outside the biosafety level 3 laboratory. The actual data acquisition is conducted within seconds, using standard ELISA plate readers present in most routine diagnostics departments. We believe that this is advantageous compared to other NT assays that rely on more sophisticated and more expensive devises, e.g., for imaging cytometry (39). The icELISA and icNT provide increased data quality and precision by generating continuous data sets. Since the detection antibodies can be applied in icELISA and icNTs in relatively high dilutions (1/5,000 to 1/10,000 and 1/2,000 of primary and secondary antibody, respectively), the assay is relatively cheap (in our case, around 0.10 € per well for both antibodies and the TMB peroxidase ELISA substrate). The specificity of the icELISA and icNT is provided by the defined SARS-CoV-2 added to the cell cultures on purpose. The primary and secondary detection antibodies just serve to visualize and quantify viral antigens. Thus, SARS-CoV-2-specific antibodies can be applied for icELISA detection notwithstanding potential cross-recognition of other CoVs such as HCoV-HKU1 or HCoV-OC43 - simply because these viruses are not present in the culture. Obviously, such antibodies recognizing conserved residues cannot be used for classic antigen-recognizing ELISAs due to their inability to discriminate coronaviruses. More virus (>1PFU/cell) can be applied to icNTs. Such high PFU icNTs scrutinize the virus-neutralizing capacity of sera more strictly, enabling a higher resolution compared to PRNT assays that all rely on low virus numbers. It is tempting to speculate that sera exhibiting superior neutralization in high PFU icNT might be more beneficial in CP therapies.

NTs based on pseudo-typed viruses using heterologous expression of the SARS-CoV-2-encoded S by non-related viruses may have certain advantages. However, genetically modified viruses are inapplicable by law in various routine diagnostics departments and usually unavailable in less developed countries. Recently, an elegant surrogate assay has been designed that determines if antibodies are capable to prevent the interaction of immobilized hACE2 with a reporter enzyme-coupled receptor binding domain (RBD) of S (40). This assay showed a good correlation with conventional NTs (r^2^ = 0.8591; p value < 0.0001) and NTs based on pseudo-particles (r^2^ = 0.8374). However, without the use of genuine infectious viruses, assays relying on pseudo-typed viruses do not fully interrogate the full spectrum of antiviral effects for example if other viral proteins influence the system, e.g., by complement activation (41). Accordingly, the icNT showed a superior correlation compared to PRNT (r^2^ = 0.987; p value 5.81E-50).

Taken together, we propose the icELISA and icNT for the quantification of SARS-CoV-2 replication and its inhibition by NAbs and antiviral compounds. By changing the detection antibody and, if necessary, the cells according to the viral infection system, the test principle is transferable to all other viruses.

## Data Availability Statement

All datasets presented in this study are included in the article/**Supplementary Material**.

## Ethics Statement

The studies involving human participants were reviewed and approved by Ethics committee of the medical faculty of the University of Duisburg- Essen (20-9208-BO, 20-9511-BO, and 20-9512-BO). The patients/participants provided their written informed consent to participate in this study.

## Author Contributions

LS, VTKL-T, ME, and DM performed research. OA, AK, and AH provided essential reagents. LS, VTKL-T, and MT analyzed the data. LS, VTKL-T, UD, and MT interpreted the data. VTKL-T and MT supervised the project. LS, VTKL-T, and MT wrote the manuscript. All authors contributed to the article and approved the submitted version.

## Funding

The authors received support by the Stiftung Universitätsmedizin Essen, Kulturstiftung Essen, the Else-Kröner Promotionskolleg ELAN, and the Deutsche Forschungsgemeinschaft (DFG) through grants RTG 1949/2, TR1208/1-1, and TR1208/2-1. The funders had no role in study design, data collection and analysis, decision to publish, or preparation of the manuscript.

## Conflict of Interest

The authors declare that the research was conducted in the absence of any commercial or financial relationships that could be construed as a potential conflict of interest.

## Supplementary Material

Supplementary Figure 1 The infectious virus dose strongly influences the neutralization capacity of serum samples. Graded amounts of SARS-CoV-2 were incubated with indicated dilutions of a serum sample (chosen to exhibit a low neutralizing capacity) for 1 h before Vero E6 cells were infected. Neutralization was evaluated by icELISA.

Supplementary Figure 2 The results of the high MOI icNT correlate with residual virus after neutralization. SARS-CoV-2 was incubated with indicated dilutions of serum samples for 1 h before Vero E6 cells were infected. **(A)** Neutralizing capacity was evaluated by icELISA. **(B)** Neutralizing capacity was evaluated by back titration of residual virus by TCID50 in 2-fold serial dilutions.

Supplementary File 1 Detailed SARS-CoV-2 icNT and icELISA laboratory protocol.
