# Supplementary figures and images for: A Novel In-Cell ELISA Assay Allows Rapid and Automated Quantification of SARS-CoV-2 to Analyze Neutralizing Antibodies and Antiviral Compounds
Source: Front Immunol. 2020 Oct 9;11:573526. doi: 10.3389/fimmu.2020.573526 (PMC7581787; doi:10.3389/fimmu.2020.573526)

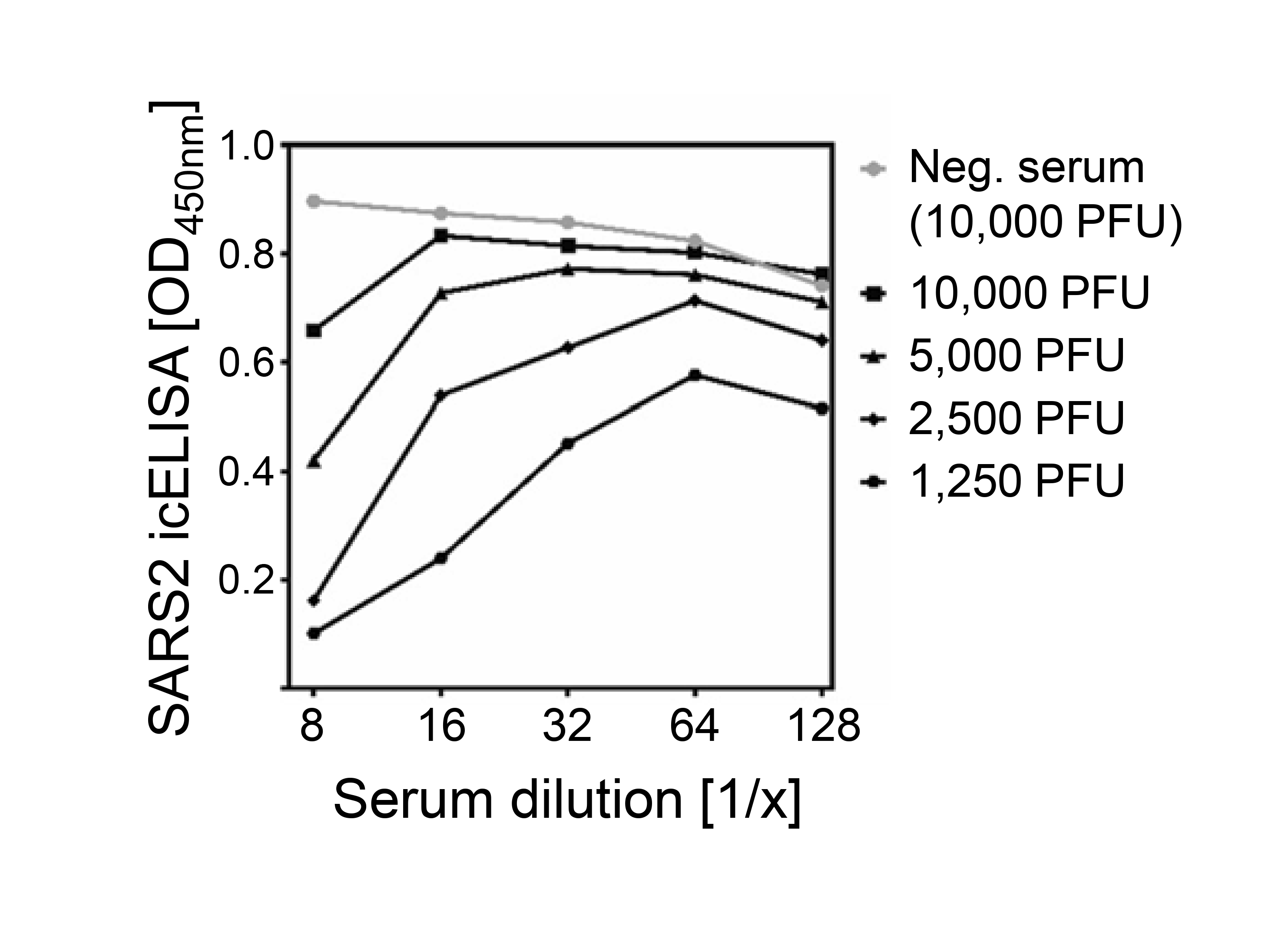

Supplement: Supplementary Figure 1 — The infectious virus dose strongly influences the neutralization capacity of serum samples. Graded amounts of SARS-CoV-2 were incubated with indicated dilutions of a serum sample (chosen to exhibit a low neutralizing capacity) for 1 h before Vero E6 cells were infected. Neutralization was evaluated by icELISA. [file Image_1.tif]

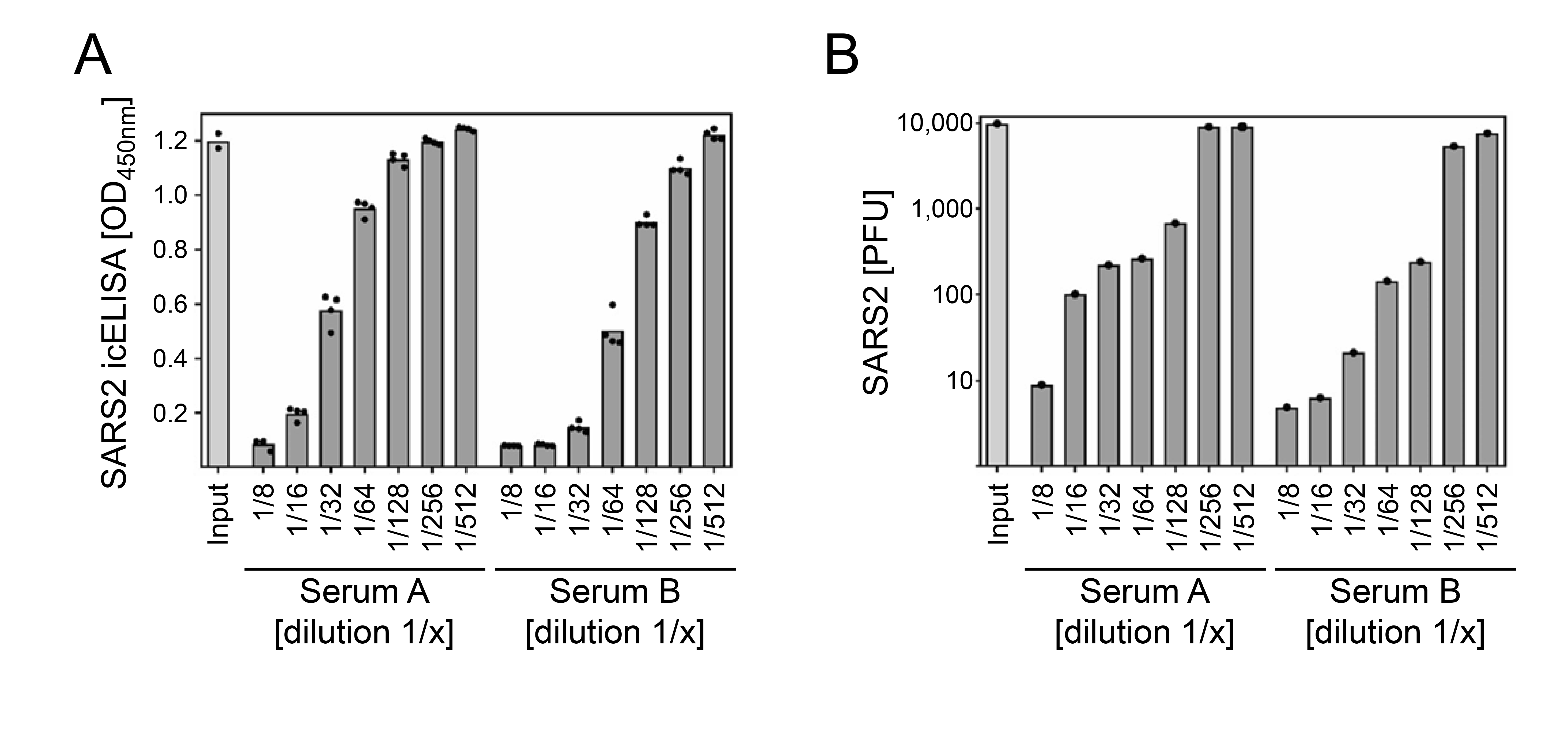

Supplement: Supplementary Figure 2 — The results of the high MOI icNT correlate with residual virus after neutralization. SARS-CoV-2 was incubated with indicated dilutions of serum samples for 1 h before Vero E6 cells were infected. (A) Neutralizing capacity was evaluated by icELISA. (B) Neutralizing capacity was evaluated by back titration of residual virus by TCID50 in 2-fold serial dilutions. [file Image_2.tif]
