# Supplementary material for: A Novel In-Cell ELISA Assay Allows Rapid and Automated Quantification of SARS-CoV-2 to Analyze Neutralizing Antibodies and Antiviral Compounds
Source: Front Immunol. 2020 Oct 9;11:573526. doi: 10.3389/fimmu.2020.573526 (PMC7581787; doi:10.3389/fimmu.2020.573526)
Supplement: Supplementary File 1 — Detailed SARS-CoV-2 icNT and icELISA laboratory protocol. [file DataSheet_1.pdf]

SARS-CoV-2 icNT (steps 1 to 13) and icELISA (steps 6 to 13)

Materials:

- Vero E6 cells
- DMEM + Glucose/Glutamine/Pyruvate supplemented with 10% FCS, Pen/Strep
- Virus stock SARS-CoV-2
- Primary and secondary antibody (e.g., α-N mAb, α-mouse IgG POD-coupled)
- Paraformaldehyde (PFA)
- 1x PBS (PBS), 2x PBS
- Triton-X-100
- Tween-20
- Fetal Calf Serum (FCS)
- Tetramethylbenzidine (TMB)
- 0.5 M HCl
- Distilled or deoinized water

Equipment:

- 96-well microplate
- 37°C CO_2 incubator
- Multichannel pipette
- Microplate reader

Notes:

- The wells of the microplate should not be allowed to dry at any point during the assay procedure.
- It is recommended to use a plate shaker during the incubation steps of the icELISA.

REAGENT	REAGENT PREPATION INSTRUCTION	WHEN TO PREPARE?
Wash Buffer	1 liter PBS + 500µl Tween-20	At start of experiment
8% (w/v) Paraformaldehyde Solution	1.) Dissolve a PBS tablet in 500 ml distilled or deionized water (= 2xPBS)<br>2.) Add 80 gr PFA<br>3.) Use 4 M NaOH to dissolve the PFA<br>4.) Use HCl for pH adjustment (pH 7,4)<br>5.) Fill up with distilled or deionized water (1 liter)	At start of experiment (store in aliquots at -20°C)
Permeabilization Buffer	For one 96-well microplate:<br>Dilute 120 µl Triton-X-100 in 12 ml PBS. Mix well by vortexing.	Prepare immediately before use
Blocking Solution	3% (v/v) FCS in PBS	Prepare immediately before use
Incubation Buffer	1% (v/v) FCS in PBS	Prepare immediately before use

icNT icELISA Trilling Lab

1.) Seed Vero E6 cells into 96-well microplates the day before.
   → Perform the test at least in duplicate, better in quadruplicate.
   → Include on every plate virus control and NT with control sera as references.
   a. Cells should be 80 - 100% confluent at the day of infection.
   b. Plates can be prepared outside of the BSL3 lab.

2.) Inactivate the serum samples at 56°C for 30 min. Dilute the control sera and the sera to be tested 1/4, 1/8, 1/16, 1/32, 1/64, 1/128, 1/256.
   → Final serum dilutions are 1/8, 1/16, 1/32, 1/64, 1/128, 1/256, 1/512 after adding the virus solution.
   → Add further dilutions if needed.
   a. Calculate the required total volume of the different dilutions (50 µl per well) including at least 20% excess volume.
   b. Prepare double the amount of the required volume of 1/4 serum dilution in medium (to do 2-fold serial dilutions).
   c. Prepare stepwise the other dilutions.
   d. Discard the excess amount of the last dilution.
   e. Serum dilutions can be prepared outside of the BSL3 lab.

3.) Prepare the virus solution (5.5 ml per 96-well microplate).
   a. Calculate the required amount of total virus (200 - 10,000 PFU per well in 50 µl).
   b. Prepare the virus solution by diluting the required amount of virus stock in medium.

4.) Neutralisation: Incubation of virus with serum.
   a. Add virus solution to the prepared serum dilutions (50 µl serum dilution + 50 µl virus solution per well).
   b. Incubate for 60 - 90 min at 37 °C.

5.) Infection.
   a. Add the virus-serum solution to the cells (max. 150 µl volume per well).
   b. Include the virus control and control sera on every plate.

6.) Fix cells to microplate.
   a. At 16 - 24 h p.i., add an equal volume of 8% paraformaldehyde solution to the wells containing culture media (= 4% paraformaldehyde solution)
   b. Incubate for at least 2 h (or overnight) at room temperature.
   → For experiments using non-BSL3 organisms, 15 min of fixation is sufficient.

7.) Replace the lids with new ones. Wipe the plates thoroughly with 4% Dismozon. Plates can now be discharged from the BSL3 laboratory.

8.) Gently aspirate the fixing solution from the microplate. Wash the microplate 3 times with 250µl PBS per well. Add 200 µl PBS to the wells. The microplate (sealed with parafilm) can now be stored at 4°C for several days.

icNT icELISA Trilling Lab

9.) Permeabilize cells.
   a. Prepare permeabilization buffer (for one microplate: dilute 120 µl Triton-X-100 in 12 ml PBS).
   b. Aspirate PBS and add 100 µl of freshly prepared permeabilization buffer to each well.
   c. Incubate for 30 min.

10.) Blocking.
   a. Aspirate permeabilization buffer and add 100 µl of blocking solution (3% FCS in PBS) to each well.
   b. Incubate for 1-2 h.

11.) Incubation with primary antibody.
   a. Prepare primary antibody (α-SARS-CoV-2-N, 1:5,000 to 1:10,000) by diluting stock antibody in the required volume of incubation buffer (1% FCS in PBS).
   b. Aspirate blocking solution and add 50 µl of antibody solution to each well.
   c. Incubate for 2 h at room temperature or overnight at 4°C (sealed with parafilm).

12.) Incubation with secondary antibody.
   a. Prepare antibody solution by diluting stock antibody in the required volume of incubation buffer (1% FCS in PBS).
   b. Aspirate primary antibody solution. Wash the microplate 3 times with 250 µl wash buffer per well.
   c. Aspirate the wash buffer and add 50 µl of secondary antibody solution to each well.
   d. Incubate for 1-2 h at room temperature.
   e. Aspirate secondary antibody solution. Wash 4 times with 250 µl wash buffer per well.

13.) Signal measurement.
   a. Prepare the required amount of TMB and 0.5 M HCl.
   b. Aspirate the last wash. Make sure that you have completely removed the liquid.
   c. Add 50 µl TMB (blue color development).
   d. Stop the reaction with 50 µl of 0.5 M HCl (by the time the uninfected wells [mock] are starting to turn blue as well).
   e. Record data at 450 nm absorbance, 620 nm reference using a microplate reader.

icNT icELISA Trilling Lab
